# Apoptosis-Associated Speck-Like Protein Containing a CARD Deletion Ameliorates Unilateral Ureteral Obstruction Induced Renal Fibrosis and Endoplasmic Reticulum Stress in Mice

**DOI:** 10.1155/2018/6909035

**Published:** 2018-07-02

**Authors:** Yao Xu, Yuqing Liu, Honglei Guo, Wei Ding

**Affiliations:** ^1^Division of Nephrology, Shanghai Ninth People's Hospital, School of Medicine, Shanghai Jiao Tong University, 639 Zhizaoju Road, Shanghai 200011, China; ^2^Division of Nephrology, The First Affiliated Hospital of Nanjing Medical University (Jiangsu Province Hospital), 300 Guangzhou Road, Nanjing, Jiangsu Province 210009, China

## Abstract

Inflammation might be one of the essential underlying mechanisms of renal fibrosis, which is considered a key pathological feature of end-stage renal disease and is closely associated with proteinuria and decreased renal function. Apoptosis-associated speck-like protein containing a CARD (ASC), identified as the central structure of inflammasome, is involved in the progression of interstitial fibrosis; however, its signal transduction pathways remain unclear. In the present study, we performed unilateral ureter obstruction (UUO) in both wild-type and ASC deletion mice to determine the contribution of ASC to renal fibrosis. Compared with control groups, UUO significantly induced renal fibrosis and collagen deposition, as evidenced by photomicrographs. ASC deletion attenuated renal injury, reduced cell infiltration and the release of inflammatory cytokines, protected against apoptosis, and downregulated the PRKR-like endoplasmic reticulum kinase (PERK) pathway of endoplasmic reticulum (ER) stress. Our data identify a novel role of ASC in the regulation of renal fibrosis and ER stress after UUO, strongly indicating that ASC could serve as an attractive target in the treatment of chronic kidney disease.

## 1. Introduction

Chronic kidney disease (CKD) is a common concern associated with high mortality and disability worldwide, which leads to much lower quality of life and a substantial economic burden [1, 2]. Renal fibrosis, closely associated with proteinuria and the decreased glomerular filtration rate (GFR) [3], is considered as a key pathological feature of end-stage renal dysfunction. Interstitial fibrosis contributes to adverse long-term renal prognosis, and inflammation might be one of the essential underlying mechanisms [4]. Inflammatory mediators, such as chemokines or cytokines, interact with myofibroblasts, recruiting more inflammatory cells that migrate into the renal interstitium, thereby causing progressive glomerulosclerosis, mesangial proliferation, and collagen deposition [5]. Despite recent rapid advances in research into CKD, the detailed pathways remain to be revealed. Such insights might lead to new therapeutic targets for CKD.

ASC (apoptosis-associated speck-like protein containing a CARD), characterized as the central structure of inflammasome, contains a pyrin domain (PYD) and a caspase recruitment domain to link pattern recognition receptors (PRRs) with procaspase 1. Consequently, prointerleukin (IL)-1*β* and pro-IL-18 are cleaved into their activated forms to trigger the downstream inflammation cascade [6]. Unsurprisingly, given its critical role under multiple pathophysiological conditions, ASC is broadly involved in the progression of CKD; however, its signal transduction pathways remain unknown [7].

The endoplasmic reticulum (ER) is responsible for modifying and folding proteins correctly, serving as a quality control system to maintain protein homeostasis [8]. Upon various endogenous disturbances and external irritants, consequent accumulation of misfolded proteins in the lumen causes ER stress and induces downstream regulation, defined as the unfolded protein response (UPR). ER stress is related to kidney diseases; while, its interaction with ASC or the inflammasome requires further research [9–11].

In this study, we focused on the role of ASC in unilateral ureter obstruction- (UUO-) induced renal fibrosis and apoptosis, tried to detect its regulation potential on ER stress or UPR. Our findings expand the understanding of the pathogenesis of CKD.

## 2. Materials and Methods

### 2.1. Materials

Antibodies recognizing the following proteins were used in this study: caspase-3, *α*-SMA, and E-cadherin, Bax, Bcl-2, BIP, ATF-4, CHOP, P-eIF2a, and eIF2a (Cell Signaling Technology); IL-1*β*, IL-18, collagen I, fibronectin, CD11b, F4/80 (Abcam); Ly6G (Protein Specialists); and caspase 1 (Santa Cruz Biotechnology, Santa Cruz, CA, USA).

### 2.2. Animal Studies

C57BL/6J (wild-type, WT) mice were purchased from Shanghai SLAC Laboratory Animals (Shanghai, China). ASC^−/−^ mice (C57BL/6J genetic background) were generated as described previously [12]. Only age- and sex-matched mice were applied in this study. All animal experiments were performed under the approval of the Animal Care Committee at Jiao Tong University. Male WT and ASC^−/−^ mice (aged 8–10 weeks) were divided equally into a control group (sham) and a model group (UUO); each group contained six mice. Complete ureteral obstruction was induced as previously described [13] on anesthetized animals. After isolation of the left kidney and ureter, the left ureter was ligated using 4–0 silk thread in the UUO group, while the ureter was simply isolated without ligation in the sham group. Mice were sacrificed at 14 days after surgery. Kidney tissues were collected and placed in 10% paraformaldehyde or immediately frozen in liquid nitrogen for storage.

### 2.3. Photomicrographs and Immunohistochemical Studies

Renal samples fixed with 10% formalin were dehydrated in alcohol and cut into 4 mm slices. Periodic acid-Schiff (PAS) and Masson trichrome staining were performed following the protocol. Specifically, the glomerular injury score was evaluated following PAS staining according to glomerulosclerosis and extracellular matrix expansion, which were scored from 0 to 4+ (0, no change; 1+, changes affecting 5–25% of the sample; 2+, changes affecting 25–50%; 3+, changes affecting 50–75%; and 4+, changes affecting 75–100%). At least 60 glomeruli were counted under microscope from 6 mice in each group, and the averaging scores were calculated as final glomerular injury score. As for tubulointerstitial fibrosis index, the areas of interstitial fibrosis were evaluated in 10 random ×400 magnification fields obtained from Masson trichrome staining. All of these observations were made by two independent researchers who were blinded to the experimental groups. The kidney samples were also stained with anticollagen I (1 : 1000) and antifibronectin antibodies (1 : 1000). The immunohistochemistry was quantified according to the method detailed in our previous study [13]. The areas of collagen I and fibronectin positivity were represented as a percentage of the total section, as analyzed using an image analyzer (Winroof; Mitani Corporation, Tokyo, Japan). The immunohistochemistry of CD11b, F4/80, and Ly6G was performed on paraffin-embedded renal tissue following protocols and imaged after incubated with secondary antibodies. Positive cells (brown pixels) were calculated from 10 high-power fields per group to indicate cell infiltration in kidney after UUO.

### 2.4. Western Blot Analysis

Protein lysates were separated by electrophoresis using 10% and 12% SDS gels. The proteins were transferred onto polyvinylidene fluoride (PVDF) membranes, which were blocked with 5% skim milk for 1 h and then incubated with primary antibodies against *α*-SMA, E-cadherin, caspase 1, IL-1*β*, IL-18, Bcl-2, Bax, caspase 3, p-eIF2*α*, ATF4, BIP, and CHOP overnight at 4°C. After washing with TBST (Tris-buffered saline Tween 20), the membranes were incubated with secondary antibodies for 1 h. Detection of the immunoreactive protein bands intensity was achieved using enhanced chemiluminescence and analyzed using Quantity One software (Bio-Rad, Hercules, CA, USA).

### 2.5. Real-Time Reverse Transcription Polymerase Chain Reaction (RT-PCR)

Total RNA was extracted from kidney tissues using TRIzol reagent (Invitrogen, Carlsbad, CA) and reverse-transcribed into cDNA following the instructions of PrimeScript RT reagent kit (Takara, Dalian, Liaoning, China). RT-PCR analysis was performed in the 7500 Fast Real-Time PCR System (Applied Biosystems, Rockford, IL, USA) as previously described, and the primer sequences were as follows: NLRP3-F, 5′ACATCTCCTTGGTCCTCAGC3′, NLRP3-R, 5′GCTTCAGTCCCACACACAGA 3′, IL-1*β*-F, 5′TTGTGGCTGTGGAGAAGCTG3′, IL-1*β*-R, 5′GCCGTCTTTCAT ACACAGG3′, IL-6-F, 5′GTGCCTCTTTGCTGCTTTCAC3′, IL-6-R, 5′GGTACATCCTCGACGGCATCT3′, IL-18-F, 5′GCTTGAATCTAAATTATCAGTC3′, IL-18-R, 5′GAAGATTCAAATTGCATCTTAT3′, TNF-*α*-F, 5′CCCTCACACTCAGATCATCTTCT3′, TNF-*α*-R, 5′GCTACGACGTGGGCTACAG3′, 18S-F, 5′TTCGGAACTGAGGCCATGATT3′, and 18S-R, 5′TTTCGCTCTGGTCCGTCTTG3′.

Relative mRNA expression was normalized to 18 S rRNA and presented as fold over control.

### 2.6. Terminal Deoxynucleotidyl Transferase-Mediated Deoxyuridine Triphosphate Nick-End Labeling (TUNEL) Assay

Apoptosis of renal tissue was detected using a TUNEL kit (Roche, Netley, NJ, USA) following the manufacturer's protocol [13]. After incubated with TUNEL reagent in dark for 60 min, 10 fields were selected randomly for each section and TUNEL-positive cells were counted and averaged.

### 2.7. Statistical Analyses

Data are expressed as the mean ± standard error of the mean (SEM). Comparisons between groups were performed using one-way analysis of variance (ANOVA) followed by Dunnett's multiple comparison tests or Student's *t*-test. *P* < 0.05 was considered statistically significant. All statistical analyses were performed using SPSS 20.0 software (IBM, Armonk, NY, USA).

## 3. Results

### 3.1. ASC Deficiency Minimized Renal Injury after UUO

**Figure 1:**
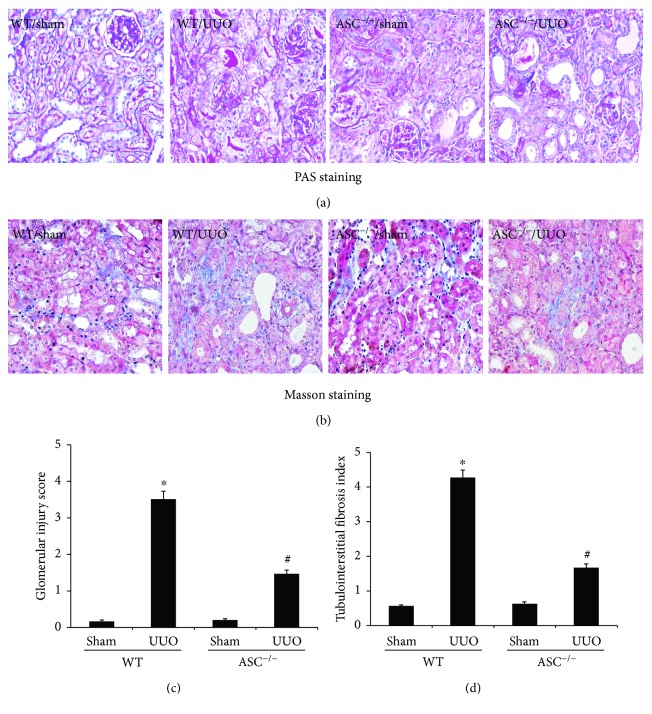
ASC deletion attenuates UUO-induced renal injury in mice. (a) Representative photomicrographs at 14 days after UUO of PAS-stained sections (magnification, ×400). (b) Masson trichrome-stained kidney sections (magnification, ×400). (c) Glomerular injury scores and (d) tubulointerstitial fibrosis indices evaluated based on PAS and Masson staining; over 20 sections for each group were counted. ^∗^*P* < 0.05, WT/sham group versus WT/UUO group. ^#^*P* < 0.05, ASC^−/−^/UUO group versus WT/UUO group. Data represent the mean ± SEM.

To determine whether ASC contributes to the progression of renal fibrosis, UUO was performed in both ASC^−/−^ mice and WT mice. After 14 days, the model groups exhibited severe tubular necrosis, epithelial degeneration, and inflammatory infiltration compared with the sham groups, while ASC deletion markedly reduced the extent of injury, as observed through PAS and Masson trichrome staining of kidney sections (Figures 1(a) and 1(b)). Additionally, ASC^−/−^ mice displayed a significantly lower glomerular injury score (1.46 ± 0.11 versus 3.51 ± 0.22) and interstitial fibrosis index (1.67 ± 0.195 versus 4.25 ± 0.518) compared with WT/UUO mice, which was in line with the pathological changes (Figures 1(c) and 1(d)). These findings indicated that UUO results in renal injury and ASC deficiency could minimize its effect *in vivo*.

### 3.2. ASC Deletion Alleviates Collagen Deposition and Renal Fibrosis

**Figure 2:**
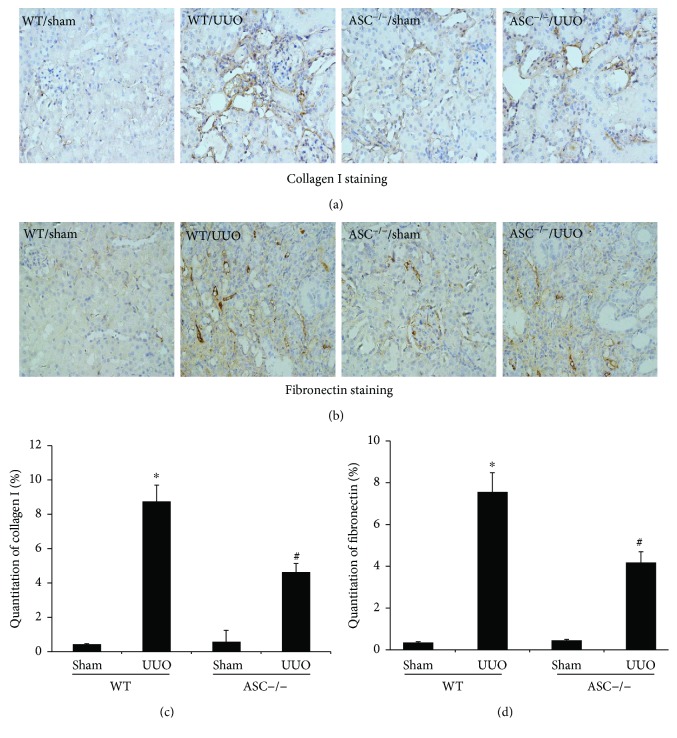
ASC deletion attenuates UUO-induced renal fibrotic cytokines in mice. (a) Representative photomicrographs at 14 days after UUO of collagen I staining (magnification, ×400). (b) Representative photomicrographs at 14 days after UUO of fibronectin staining (magnification, ×400). (c) Semiquantitative analysis of collagen I among the different groups. (d) Quantification of fibronectin-positive areas. ^∗^*P* < 0.05, WT/sham group versus WT/UUO group. ^#^*P* < 0.05, ASC^−/−^/UUO group versus WT/UUO group. Data represent the mean ± SEM (*n* = 6).

Previous studies showed that activation of profibrotic mediators, such as collagen (especially collagen I) and fibronectin, is the key feature of interstitial fibrosis in CKD [14]. Therefore, we detected collagen I and fibronectin deposition in renal tissue from the ligated site using immunohistochemical staining. A dramatic increase in collagen I (Figure 2(a)) and fibronectin-positive cells (Figure 2(b)) in WT mice was observed at 14 days after UUO. However, semiquantitative analysis demonstrated a marked reduction in collagen I (4.62 ± 0.51 versus 8.73 ± 0.96, *P* < 0.05) and fibronectin (4.17 ± 0.53 versus 7.55 ± 0.92) deposition in ASC deletion mice compared with WT/UUO group (Figures 2(c) and 2(d)).

### 3.3. ASC Regulates UUO-Induced Phenotypic Alterations and Apoptosis in Renal Fibrosis

**Figure 3:**
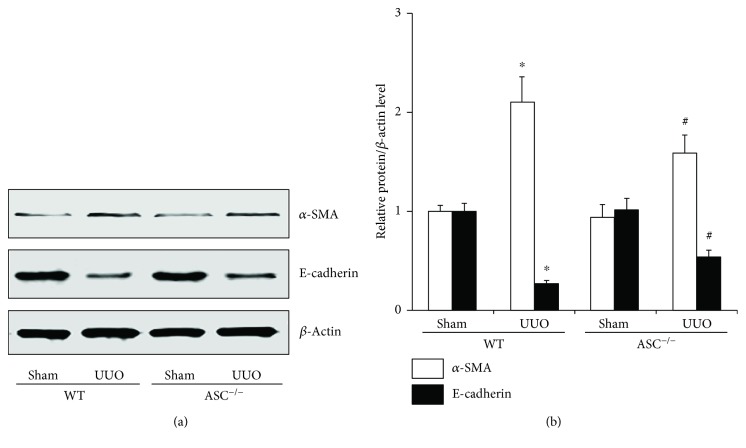
ASC regulates phenotypic alterations in UUO mice. (a) Immunoblotting for *α*-SMA and E-cadherin in WT and ASC^−/−^ mice with or without UUO after 2 weeks. (b) Quantification of *α*-SMA and E-cadherin levels, normalized against those of *β*-actin. ^∗^*P* < 0.05, WT/sham group versus WT/UUO group. ^#^*P* < 0.05, ASC^−/−^/UUO group versus WT/UUO group. Data represent the mean ± SEM (*n* = 6).

During phenotypic alterations, tubular cells typically lose their epithelial characteristics and express protein characteristic of myofibroblasts. To further unravel the regulatory effect of ASC in this process, *α*-smooth muscle actin (*α*-SMA) and E-cadherin were chosen as the phenotypic change markers. Compared with the sham operation groups, UUO led to a remarkable increase in *α*-SMA (2.1-fold, WT/UUO) expression and a decrease in E-cadherin (0.268-fold, WT/UUO). Interestingly, ASC^−/−^ mice exhibited a partial inhibition of tubular cell epithelial-mesenchymal transition (EMT), represented as relatively higher E-cadherin level and less *α*-SMA (Figure 3).

**Figure 4:**
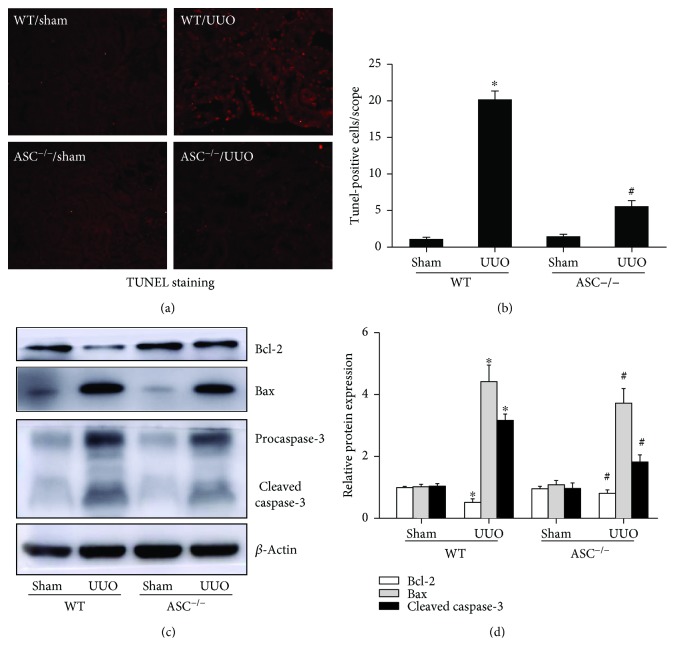
ASC^−/−^ protects against apoptosis associated with UUO. (a) Images of TUNEL staining (magnification, ×200). (b) Average number of TUNEL-positive cells per scope, 10 fields were selected randomly over 6 mice for each group. (c) Immunoblotting for Bcl-2, Bax, and caspase 3 protein expression at day 14 after UUO. (d) Quantification of Bcl-2, Bax, and cleaved caspase 3 levels, normalized against those of *β*-actin. ^∗^*P* < 0.05, WT/sham group versus WT/UUO group. ^#^*P* < 0.05, ASC^−/−^/UUO group versus WT/UUO group. Data represent the mean ± SEM (*n* = 6).

In addition, Western blotting analysis of Bcl-2 and Bax demonstrated that in UUO mice, the level of Bcl-2 was downregulated. By contrast, Bax, a common marker of apoptosis, was significantly upregulated and, in turn, caused a prominent increase in cleaved caspase 3 (Figures 4(c) and 4(d)). Importantly, ASC deletion consistently decreased the level of Bax and caspase 3. TUNEL staining also confirmed that the apoptosis induced by UUO was markedly rescued by ASC depletion, which indicated that ASC^−/−^ protected against cell apoptosis in the UUO model of CKD (Figures 4(a) and 4(b)).

### 3.4. ASC^−/−^ Mice Display Reduced Inflammation after UUO

**Figure 5:**
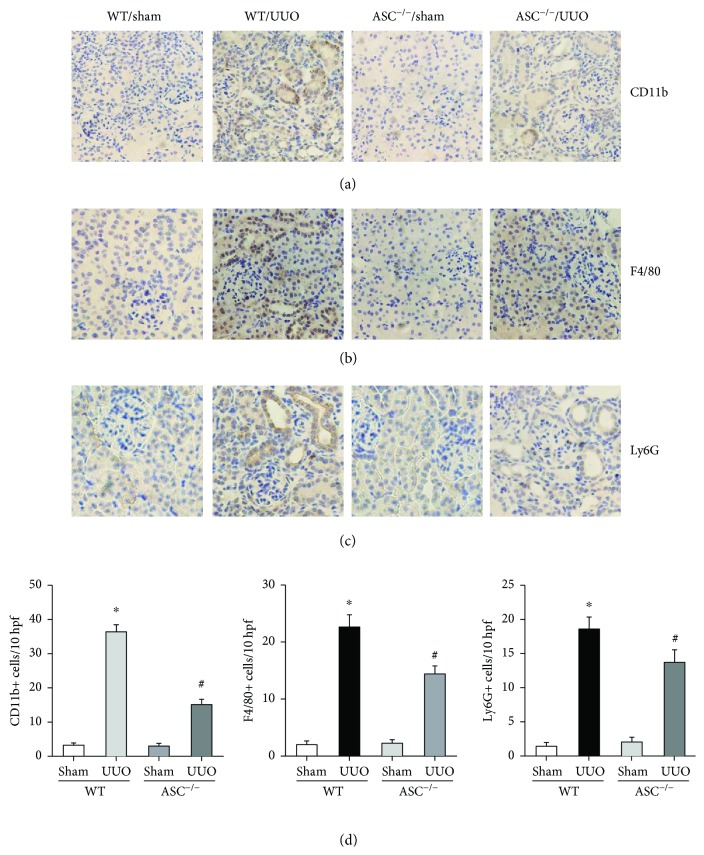
Renal inflammatory cell infiltration after UUO is reduced in ASC^−/−^ mice. Immunohistochemistry (magnification, ×400) of (a) CD11b-positive leukocytes, (b) F4/80-positive macrophages, and (c) Ly6G-positive granulocytes. (d) Quantification of inflammatory cell infiltration (brown pixels) in renal interstitium. ^∗^*P* < 0.05, WT/sham group versus WT/UUO group. ^#^*P* < 0.05, ASC^−/−^/UUO group versus WT/UUO group. Data represent the mean ± SEM (*n* = 10).

Inflammation is integrally associated with renal interstitial fibrosis; therefore, immunohistochemistry was performed to detect the influence of ASC deletion on inflammatory cell infiltration and whether it was connected with the restored renal function. Consistent with pathological changes, UUO mice appeared severely aggravated immune cell infiltration of CD11b-positive leukocytes, F4/80-positive macrophages, and Ly6G-positive granulocytes in renal interstitium at 14 days (Figure 5). Quantification analysis confirmed that absence of ASC significantly reduced the cellular infiltration mentioned above, compared with wild-type controls.

**Figure 6:**
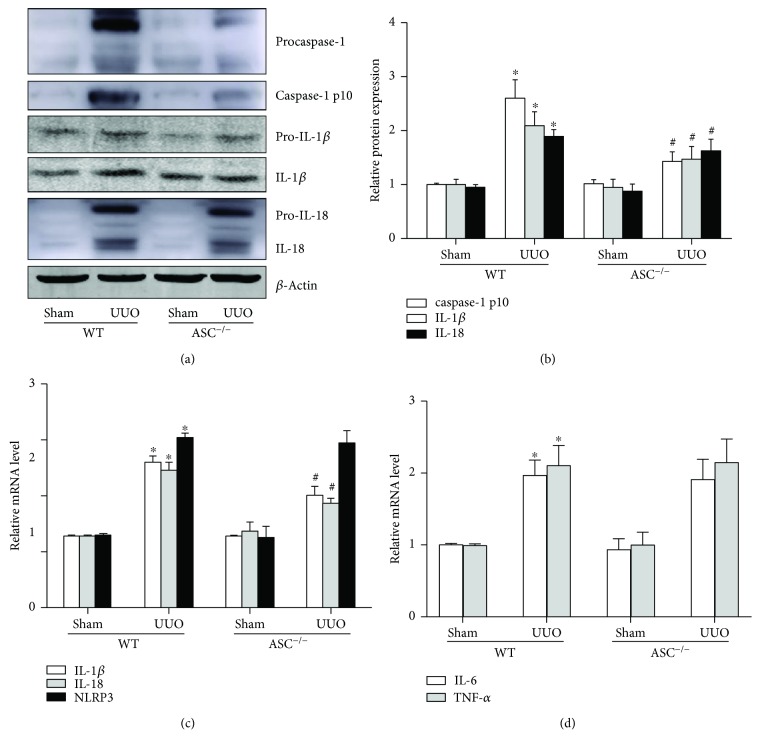
ASC deletion reduces the expression of cytokines that are upregulated by inflammasomes in the UUO model of CKD. (a) Immunoblotting for the level of caspase 1, IL-18, and IL-1*β*. (b) Quantification analysis of caspase 1, IL-18, and IL-1*β* levels, normalized against those of *β*-actin. Semiquantitative analysis of NLRP3, IL-18, and IL-1*β* (c) and IL-6 and TNF-*α* gene expression (d) normalized against 18S performed by real-time PCR. ^∗^*P* < 0.05, WT/sham group versus WT/UUO group. ^#^*P* < 0.05, ASC^−/−^/UUO group versus WT/UUO group. Data represent the mean ± SEM (*n* = 6).

Since ASC serves as central functional component of inflammasome, cytokines such as IL-18 and IL-1*β* should be involved in the regulation process. Therefore, immunoblotting for the proinflammatory mediators was performed. In line with our hypothesis, the levels of caspase 1, cleaved IL-18, and IL-1*β* were markedly increased in the UUO groups compared with the sham controls. ASC deletion downregulated the level of caspase 1 and dramatically inhibited the expression and maturation of IL-18 and IL-1*β*, providing further evidence that ASC modulates inflammation during the disease process (Figures 6(a) and 6(b)). Similarly, gene expressions of NLRP3, IL-18, and IL-1*β* were upregulated after UUO. ASC^−/−^ mice showed decreased mRNA levels of IL-18 and IL-1*β* but not NLRP3. However, the expression of other cytokines like IL-6 and TNF-*α* was not affected by ASC knockout as presented by RT-PCR (Figures 6(c) and 6(d)).

### 3.5. ASC Is a Regulator of the ER Stress Pathway

**Figure 7:**
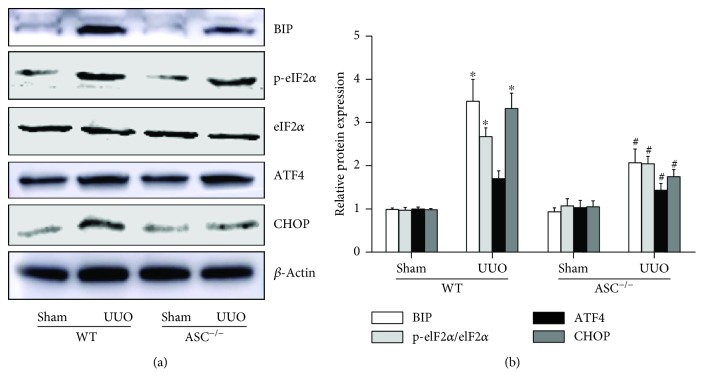
Activation of ER stress induced by UUO is inhibited by ASC deficiency. (a) Immunoblotting for the levels of BIP, p-eIF2*α*/eIF2*α*, ATF4, and CHOP. (b) Quantification analysis of BIP, p-eIF2*α*/eIF2*α*, ATF4, and CHOP levels, normalized against those of *β*-actin. ^∗^*P* < 0.05, WT/sham group versus WT/UUO group. ^#^*P* < 0.05, ASC^−/−^/UUO group versus WT/UUO group. Data represent the mean ± SEM (*n* = 6).

ASC and ER stress are both closely related to renal tubulointerstitial fibrosis, which prompted us to investigate the linkage between ASC and ER stress in UUO-induced CKD. To determine the possible underlying mechanism, the activation of CHOP, an ER stress marker, was evaluated. As shown by Western blotting analysis, CHOP and BIP were significantly increased at day 14 after UUO in the WT mice and the ASC^−/−^/UUO mice showed significantly reduced CHOP and BIP levels. We then examined the PRKR-like endoplasmic reticulum kinase (PERK) pathway, one of the typical downstream pathways of ER stress and UPR. Similar to CHOP, the levels of both p-eIF2*α*/eIF2*α* and ATF4 increased markedly upon UUO stimulation, while the ASC^−/−^ mice presented a much lower increase compared with that in the WT/UUO group (Figures 7(a) and 7(b)). Taken together, these data revealed that the prolonged activation of ER stress and/or UPR following UUO was inhibited by ASC deletion.

## 4. Discussion

In the current study, we identified ASC as a regulator that interacts with ER stress in the progression of renal fibrosis after UUO. ASC deficiency attenuated renal injury, decreased collagen deposition, reduced cellular infiltration, and downregulated inflammasome-activated cytokines associated with ER stress and UPR activation. Our study uncovered a new layer of the complex mechanisms underlying renal fibrosis and shed light on a potential therapeutic target for CKD.

During fibrinogenesis, upon the gradual nephron loss and suspended tissue remodeling, the injured tubular cells undergo apoptosis and the capillaries become pathologically permeable, thus triggering chronic interstitial inflammation. The activation of inflammasomes, especially the maturation and secretion of cytokines, are widely involved in renal fibrosis [15]. Vesey et al. revealed that IL-1*β* could stimulate human renal fibroblast proliferation and fibronectin production and induce proximal tubule cell injury [16]. Similarly, IL-18 neutralization ameliorated UUO-induced EMT and renal fibrosis [17]. In our study, we confirmed that the infiltration of leukocytes and F4/80+ macrophages was induced by UUO. And the expression and maturation of IL-1*β* and IL-18 were markedly increased in mice at day 14, which supported the results of previous studies. By contrast, ASC knockdown reversed this phenotype to some extent, prompting us to summarize that the protective effect of ASC deletion against renal injury and fibrosis functions partially through the reduction of inflammatory mediators. Notably, the translation of pro-IL-1*β* or IL-18 and the processing of cleaved cytokines via inflammasome are separated [18]. Our data showed the effects of ASC appeared on both transcripts and proforms evidenced by PCR and Western blot, which suggested that the regulation of ASC^−/−^ on inflammation might be mostly through the inflammasome-independent pathway.

ASC is the major component of the inflammasome protein complex, which relocates into a cytosolic speck and collaborates with caspase 1 upon various PRRs stimuli. Among them, NLRP3 is the best characterized so far. NLRP3 deletion mice displayed less tubular injury, renal inflammation, and fibrosis 14 days after UUO [15]. In humans, NLRP3 expression was increased in renal biopsy samples from patients with CKD [19]. Our previous study also demonstrated that NLRP3^−/−^ significantly decreased cytokine release and preserved kidney ultrastructure in glomerular injury and tubulointerstitial fibrosis [13]. Interestingly, other studies reported contradictory results. Pulskens et al. determined that Nlrp3 knockout mice developed improved early tubular damage and interstitial edema after UUO; however, no statistical difference was found for the level of renal fibrosis [20]. Furthermore, the protective effect of NLRP3 in the early stage in UUO mice was not reversed in the later phase. In the present study, marked glomerular injury and tubulointerstitial fibrosis were observed at day 14 after UUO according to PAS and Masson staining. ASC deletion preserved kidney function via decreasing collagen deposition and reducing epithelial EMT and apoptosis, as shown by immunohistochemistry, TUNEL staining, and Western blotting analyses. Meanwhile, the regulation of inflammatory cytokines was associated with the protective phenotype in ASC knockdown mice. Interestingly, the levels of IL-6 and TNF-*α* remained stable in ASC^−/−^ mice, which suggested extensive pathways underlying inflammation after UUO independently of the assembly of NLRP3 inflammasome. Taken together, these data indicate that the role of ASC in renal fibrosis is specific for IL-1*β* and IL-18, which are known to be processed by the inflammasome, though our evidence does not fully support the conclusion that this effect is inflammasome-dependent.

Additionally, ASC could also act independent of NLRP3 or caspase 1. ASC affected MAPK phosphorylation by pathogens and Toll-like receptor agonists, distinct from inflammasome activation [21]. In multiple disease models, the epigenetic regulation of ASC/TMS1 expression was critical in the function of major host defense systems, cellular housekeeping, and carcinogenesis [22]. Ippagunta et al. also determined an independent role of ASC in T cell priming for collagen-induced arthritis [23]. Similarly, Wang et al. showed that NLRP3 promoted TGF-*β* signaling, R-Smad activation, and EMT in the kidney renal tubular epithelium. Both NLRP3^−/−^ and ASC^−/−^ would impair the pathway and reduce the expression of related genes in tubular cells, which occurred independently of caspase 1 or inflammasome-regulated cytokines [24]. Overall, the regulation of ASC in renal fibrosis must be tightly controlled and complex. More comprehensive investigations on the underlying mechanisms are required.

ER stress serves as a protective process that enhances the degradation of unfolded proteins and promotes the efficiency of correct folding to maintain protein homeostasis. However, under pathological insults, such as ischemia, infection, hypoxia, and reactive oxygen species (ROS) overload, persistent ER stress leads to ER dysfunction and the activation of the UPR. Numerous data have already identified ER stress as a key factor in tubular epithelial cell atrophy and interstitial fibrosis [25]. ER stress markers, including chaperones and CHOP, were increased in kidney biopsy samples of patients suffering from various kidney diseases [26]. CHOP deletion inhibited the UPR and attenuated aldosterone-induced interstitial fibrosis and apoptosis [27]. Our study also provided evidence of the regulation of ER stress in renal injury through ASC. CHOP was significantly increased in UUO mice, associated with elevated EMT and matrix expansion, while this effect was markedly ameliorated by ASC knockdown. Furthermore, we explored the expression of related pathways to detect the underlying mechanisms.

There are three main protein sensors that prime ER stress, ATF6, IRE1*α*, and PERK. These sensors bind to BiP in physiological conditions and dissociate after stimulation, thus activating downstream signaling, including inhibition of translation, induction of apoptosis, and ER-associated degradation [28]. In our UUO mouse model, we evaluated the levels of the PERK pathway involving eukaryotic translation initiation factor 2*α* (eIF2*α*), ATF4, and CHOP. Compared with the WT, ASC^−/−^ mice exhibited dramatically lower levels of p-eIF2*α* and ATF4, which were upregulated by UUO. ER stress-related apoptosis was also reduced. Interestingly, we also detected changes in Bcl-2, Bax, and caspase 3 levels induced by ASC deletion. The death receptor pathway, ER pathway, and mitochondrial pathway are known as the three major pathways of cell apoptosis; therefore, the role of mitochondria cannot be excluded. Consistent with our previous study, mitochondrial dysfunction contributed to renal fibrosis and chronic interstitial inflammation [19]. Similarly, recent reports elaborated an NLRP3-caspase-1-dependent mechanism that relayed ER stress to the mitochondria to promote inflammation [29]. However, the crosstalk between ER and mitochondria remains elusive and requires a more detailed investigation.

In conclusion, our study established an axis of ASC inflammation-ER stress in renal fibrosis and apoptosis. ASC deletion attenuates renal injury, extracellular matrix deposition, and tubular EMT and reduces cellular infiltration and the release of inflammatory cytokines, which are closely related to the regulation of ER stress. Our data strongly support the hypothesis that ASC could serve as an attractive target in the treatment of renal fibrosis in CKD.

## Data Availability

No additional data are available.
